# *Dunaliella salina*-Loaded Diosmetin Carriers Alleviate Oxidative Stress and Inflammation in Cisplatin-Induced Acute Kidney Injury via PI3K/AKT Pathway

**DOI:** 10.3390/pharmaceutics18010102

**Published:** 2026-01-12

**Authors:** Yujing Huangfu, Wei Chen, Dandan Guo, Peiyao Wang, Aifang Li, Yi Yang, Shuxuan Li, Qianfang Wang, Baiyan Wang, Shuying Feng

**Affiliations:** 1 Medical College, Henan University of Chinese Medicine, Zhengzhou 450046, China; 2022702005@hactcm.edu.cn (Y.H.); chenwei@hactcm.edu.cn (W.C.); gdd461900959@hactcm.edu.cn (D.G.); 2024156036@hactcm.edu.cn (P.W.); liaifang-2020@hactcm.edu.cn (A.L.); yangyi@hactcm.edu.cn (Y.Y.); lishuxuan@hactcm.edu.cn (S.L.); 2025702004@hactcm.edu.cn (Q.W.); baiyanw@hactcm.edu.cn (B.W.); 2 Henan Engineering Research Center for Chinese Medicine Foods for Special Medical Purpose, Zhengzhou 450046, China

**Keywords:** drug loading, *D. salina* delivery system, diosmetin, PI3K/AKT pathway, Inflammation, oxidative stress

## Abstract

**Background:** As a widely used chemotherapeutic agent, cisplatin frequently induces acute kidney injury (AKI), which severely compromises patient survival and limits its clinical use. While the natural flavonoid diosmetin (Dio) shows promise in mitigating cisplatin-induced nephrotoxicity, its clinical translation is challenged by poor solubility, low bioavailability, and incompletely elucidated mechanisms. This study aimed to overcome these limitations by developing a novel drug delivery system using the microalgae *Dunaliella salina* (*D. salina*, *Ds*) to load Dio (*Ds*-Dio), thereby enhancing its efficacy and exploring its therapeutic potential. **Methods:** We first characterized the physicochemical properties of *Ds* and Dio, and then *Ds*-Dio complex was synthesized via co-incubation. Its nephroprotective efficacy and safety were systematically evaluated in a cisplatin-induced mouse AKI model by assessing renal function (serum creatinine, blood urea nitrogen), injury biomarkers, histopathology, body weight, and organ index. The underlying mechanism was predicted by network pharmacology and subsequently validated experimentally. **Results:** The novel *Ds*-Dio delivery system has been successfully established. In the AKI model, *Ds*-Dio significantly improved renal function and exhibited a superior protective effect over Dio alone; this benefit is attributed to the enhanced bioavailability provided by *Ds* carrier. In addition, *Ds*-Dio also demonstrated safety performance, with no evidence of toxicity to major organs. Network pharmacology analysis predicted the involvement of PI3K/AKT pathway, which was experimentally verified. Specifically, we confirmed that *Ds*-Dio alleviates AKI by modulating the PI3K/AKT pathway, resulting in concurrent suppression of NF-κB-mediated inflammation and activation of NRF2-dependent antioxidant responses. **Conclusions:** This study successfully developed a microalgae-based drug delivery system, *Ds*-Dio, which significantly enhances the nephroprotective efficacy of Dio against cisplatin-induced AKI. The nephroprotective mechanism is associated with modulation of the PI3K/AKT pathway, resulting in the simultaneous attenuation of oxidative stress and inflammation.

## 1. Introduction

Acute kidney injury (AKI) is a clinical syndrome characterized by markedly elevated blood urea nitrogen and creatinine levels, leading to rapid deterioration or impairment of renal function [1]. Among the multiple factors that may trigger to AKI, exposure to antitumor drugs cisplatin (Cis) is one of the most common factors [2]. Current clinical management of AKI primarily involves symptomatic treatment, supportive care, and renal replacement therapy. But these approaches have limited efficacy, such as the clinical shortage of safe and effective therapeutic options [3]. Accumulating evidence suggests that cisplatin-induced acute kidney injury (Cis-AKI) involves oxidative stress, inflammation, and apoptosis [4]. Certain traditional Chinese medicine (TCM) components with antioxidant and anti-inflammatory properties show promise in treating AKI [5]. Diosmetin (Dio) is a natural flavonoid found in various plants, some of which—including rehmannia, chrysanthemum, mint, and scutellaria baicalensis—are important herbs in TCM, exhibiting anti-inflammatory, antioxidant, and antitumor activities [6,7]. Meanwhile, our preliminary data has indicated that Dio can suppress esophageal squamous cell carcinoma progression and alleviate cisplatin-induced nephrotoxicity [8], supporting its potential use in Cis-AKI treatment. However, the exact protective mechanisms of Dio against Cis-AKI remain unclear. As a flavonoid compound, the development and clinical application of Dio is limited by its poor water solubility and low bioavailability [7]. Nanoparticle-based delivery systems have emerged as a promising approach to improve drug encapsulation and delivery, potentially enhancing therapeutic efficacy [9,10,11]. However, the clinical translation of such nanoplatforms remains challenging due to the complexity of manufacturing processes, inadequate biocompatibility, and suboptimal pharmacokinetic properties [10]. Therefore, developing novel drug delivery systems that are highly biocompatible and minimally toxic is essential to improve the bioavailability of Dio. Microalgae, as a natural biological resource, representing a promising drug carrier for enhancing therapeutic outcomes by improving bioavailability and reducing side effects. Species such as *Spirulina*, *Chlorella*, and *Chlamydomonas reinhardtii* have been widely explored for this purpose [12,13,14,15]. Advanced strategies including electrostatic adsorption, surface modification, hydrogel encapsulation, and cell membrane coating have markedly enhanced drug delivery efficiency [16,17,18,19,20]. *Dunaliella salina* (*D. salina*, *Ds*), an FDA-approved microalga for food use, is abundant in bioactive compounds like β-carotene, fatty acids, and polysaccharides, which confer antioxidant, anti-inflammatory, antimicrobial, antiviral, and antitumor properties [21,22,23,24,25]. These unique properties contribute *Ds* as a promising drug delivery carrier, capable of enhancing the bioavailability of loaded drugs and exerting synergistic therapeutic effects.

In this study, we employed electrostatic adsorption to load Dio onto *Ds*, forming a drug delivery system aimed at enhancing the bioavailability of Dio. The resulting *Ds-*Dio complex was evaluated in a mouse model of cisplatin-induced AKI, where it demonstrated significant therapeutic efficacy. Using network pharmacology, we further investigated the mechanistic basis of this enhanced protection. Our findings indicate that *Ds* and Dio act synergistically to attenuate oxidative stress and suppress inflammatory responses primarily via the PI3K/AKT signaling pathway. It underlines the promise of microalgae-based drug delivery systems in enhancing the potency of flavonoid-based therapies for renal protection.

## 2. Materials and Methods

### 2.1. Regents

Dio powder was purchased from Aladdin Biology Technology Institute (Shanghai, China). Serum creatinine (Scr) and blood urea nitrogen (BUN) test kits were purchased from Nanjing Jiancheng Biological Company (Nanjing, China). AKT (cat. R23412), p-AKT (cat. R381555), NF-κb p65 (cat. 380172), p-p65 (cat. 310013) antibodies were purchased from Zen-Bioscience (Chengdu, China). NRF2 (cat. 16396-1), GPX4(cat. 67763-1), and β-actin (cat. 20536-1) antibodies were purchased from Proteintech (Wuhan, China).

### 2.2. Characterization Methods of Ds and Ds FDP

The living *Ds* cells were cultured in PKS medium at 26 °C in a lighted environment [26]. To prepare the freeze-dried powder of *Ds* (*Ds* FDP), *Ds* sample was first washed three times with ultrapure water, and then spun at 1500× *g* for 5 min. The collected *Ds* was frozen at −20 °C for 4 h, and was subsequently freeze-dried in a Labconco portable freeze-dryer (Labconco Corporation, Kansas, MO, USA). Once *Ds* and *Ds* FDP were ready, the morphology and structure of *Ds* were captured with a fluorescence microscope, scanning electron microscopy (SEM), and transmission electron microscopy (TEM). The absorption spectra of the *Ds* and *Ds* FDP were determined by ultraviolet–visible spectrophotometry, and the zeta potential of *Ds* and the *Ds* FDP were measured using the Nano-ZS instrument (Malvern Instruments Ltd., Worcestershire, UK), along with the particle size of *Ds* FDP.

### 2.3. Preparation of Ds-Dio

Dio loading was initiated when the *Ds* culture reached the logarithmic growth phase, at a cell density of approximately 5 × 10^6^ cells/mL. To determine the safe concentration of Dio for *Ds*, 10 μL of Dio at varying concentrations (0, 0.3125, 0.625, 1.25, 2.5, 5, 10 mg/mL, dissolved in DMSO) were added to 10 mL of *Ds* suspension. Following a 24 h co-culture process, the viability of *Ds* was assessed microscopically, and the number of live cells was counted. For synthesis of *Ds*-Dio, 10 μL Dio with a series of concentrations were added to 10 mL of *Ds* or *Ds* FDP suspension, respectively. The mixtures were co-cultured by stirring for 2 h at 120 rpm in a 26 °C environment. Then, the *Ds*-Dio and supernatants were collected by centrifugation at 3000× *g* for 30 min. The amount of loaded Dio was quantified by absorbance of the collected supernatants by HPLC, in combination with a standard curve of Dio. Subsequently, the drug loading efficiency was defined as following: the amount of Dio in initial solution—the amount of loaded Dio in supernatants/the amount of Dio in initial solution × 100%. The *Ds*-Dio precipitates were resuspended in PBS, and the ζ potentials of *Ds*-Dio were measured via a dynamic light scattering (DLS, Malvern, Worcestershire, UK) particle-size analyzer.

### 2.4. AKI Mouse Experiment

All animal experimental protocols were approved by the Animal Experiment Ethics Committee of Henan University of Chinese Medicine (No.2853, approved on 13 June 2025). Eight-week-old male C57BL/6 mice were procured from Beijing HFK Bioscience (Beijing, China) and subsequently housed within the barrier system. Following a one-week acclimatization period, the subjects were randomly divided into five groups, including the negative control group, model group, Dio group (50 mg/kg), *Ds* group (number of *Ds* loaded with an equivalent dose of Dio), and the *Ds*-Dio group. The Dio, *Ds*, and *Ds*-Dio groups were administered intraperitoneally via injections for a period of seven days in advance, while the other groups were given an equal volume of solvent sodium carboxyl methyl cellulose. After 7 d, AKI models were established in mice via intraperitoneal injection of cisplatin (20 mg/kg), whilst control mice received intraperitoneal injections of PBS. Treatment was continued 24 h after model establishment, with all experimental mice euthanized 48 h post-modeling. The body weight of subjects was measured daily. Blood and kidney tissue samples were collected for subsequent analysis. Furthermore, organs were collected and the organ index was calculated using the following formula: organ weight/mouse body weight × 100%.

### 2.5. Assessment of Kidney Function

The levels of Scr and BUN were determined in order to assess renal function. Blood samples of mice were left at room temperature for 2 h, and then the samples were centrifuged at 1160× *g* for 20 min at 4 °C to harvest serum. The levels of Scr and BUN were measured according to the manufacturer’s instructions, and the absorbance at 546 nm and 640 nm were recorded.

### 2.6. Quantitative Real-Time PCR

Total RNA was extracted from mice kidney tissues using TRIzol reagent (Vazyme Biotech, Nanjing, China). Subsequently, the RNA was reversed to cDNA using the Swescript RT I First Strand cDNA Synthesis Kit (Accurate Biology, Changsha, China). Quantitative real-time PCR analysis was conducted by SYBR Green (Vazyme Biotech, Nanjing, China). The relative mRNA expression was calculated by the 2^(−ΔΔCT)^ method, and GAPDH was used as an internal reference. The primer sequences of AKI-related factors (NGAL, KIM-1), inflammatory factors (IL-1β, TNFα, IL-4, IL-10), and oxidative stress markers (SOD1, HO1) are listed in Table S1.

### 2.7. Histological Analysis

The collected tissues were fixed in 4% paraformaldehyde for 24 h, after which they were paraffin-embedded and sectioned. Following dewaxing and dehydration, the sections were stained using hematoxylin and eosin (HE) and periodic acid-Schiff (PAS). Pathological changes in the organs were then examined and visualized using microscopy. The degree of kidney injury was evaluated based on cellular lysis, tubular dilation, loss of tubular brush borders, and cast formation [27]. The degree of kidney injury was graded on a scale of 0 to 4: 0 (no damage), 1 (<25%), 2 (25–50%), 3 (50–75%), and 4 (>75%) [28]. Three randomly selected tissue samples per group were analyzed. From each sample, eight random microscopic fields (×400 magnification) were assessed. Three independent pathologists performed blinded scoring based on the established criteria for glomerular and tubular injury outlined in our manuscript. The final score for each sample was derived from the average of the scores from all pathologists and fields prior to statistical analysis.

### 2.8. Network Pharmacology Analysis and Molecular Docking

The potential target data of Dio was carried out via the Swiss Target Prediction database (http://www.swisstargetprediction.ch/, 18 July 2025). The keyword ‘acute kidney injury’ was used to search both the Gene Cards database (https://www.genecards.org/, 18 July 2025) and OMIM (https://www.omim.org/, 18 July 2025) database. The Evenn website (https://www.bic.ac.cn/EVenn/#/, 15 September 2025) was utilized to generate a Venn diagram of Dio-related targets and AKI-related targets for the purpose of assessing the overlap. Subsequently, the intersection of genes was imported into DAVID database (https://davidbioinformatics.nih.gov/, 15 September 2025) for GO analysis, KEGG analysis, and Reactome analysis. GO analysis encompassed biological processes (BPs), cellular components (CCs), and molecular functions (MFs). Following this, the common targets obtained were imported into the STRING database (https://cn.string-db.org/, 16 September 2025) to construct a protein–protein interaction (PPI) network. Cytoscape software (version 3.10.3 and 3.7.0; Institute for Systems Biology, Seattle, WA, USA) was employed to optimize the visualization of these potential interaction relationships. The Centiscape 2.2 plugin was used to screen for core targets, and a total of 13 core targets were obtained. Molecular docking was performed to investigate the binding potential of Diosmetin (PubChem ID: 5281612) to AKT isoforms AKT1 (PDB ID: 1UNQ), AKT2 (PDB ID: 1O6L), and AKT3 (PDB ID: 8ZXW). The crystal structures of AKT isoforms were retrieved from the Protein Data Bank database. The protein structure was prepared with the AutoDockTools (version 1.5.7; the Scripps Research Institute, La Jolla, CA, USA), which involved the removal of water molecules and the addition of hydrogen atoms. Finally, Dio was docked into the AKT binding site using the AutoDock Vina (version 1.1.2; the Scripps Research Institute, La Jolla, CA, USA) to complete the molecular docking.

### 2.9. Western Blotting

Total protein from kidney tissues were extracted by RIPA lysis buffer (Beyotime Biotechnology, Shanghai, China) and quantified using the BCA assay kit (Beyotime Biotechnology, Shanghai, China). The proteins were then separated by SDS-PAGE and transferred to the PVDF membrane. After blocking with BSA, the membrane was incubated with different primary antibodies at 4 °C overnight. The next day, the membrane was incubated with the indicated secondary antibodies and visualized using the chemiluminescence detection kit (Beyotime Biotechnology, Shanghai, China). The signals were then quantified using Image J software (version 1.8.0; National Institutes of Health, Bethesda, MD, USA). For analysis, normalization of each sample to β-actin was performed prior to quantitative comparison.

### 2.10. Statistical Analysis

The data were expressed as means ± standard deviation (SD) and analyzed using GraphPad Prism software (version 9.0.0; GraphPad Software, Boston, MA, USA). All experiments were performed three times independently. Statistical differences were evaluated using Student’s *t*-tests or two-way ANOVA. The significance of the results in this study was denoted as follows: * indicates *p* < 0.05, ** indicates *p* < 0.01, *** indicates *p* < 0.001, **** indicates *p* < 0.0001, ^##^ indicates *p* < 0.01, and ^####^ indicates *p* < 0.0001.

## 3. Results

### 3.1. Characterization of Ds and Ds FDP

**Figure 1:**
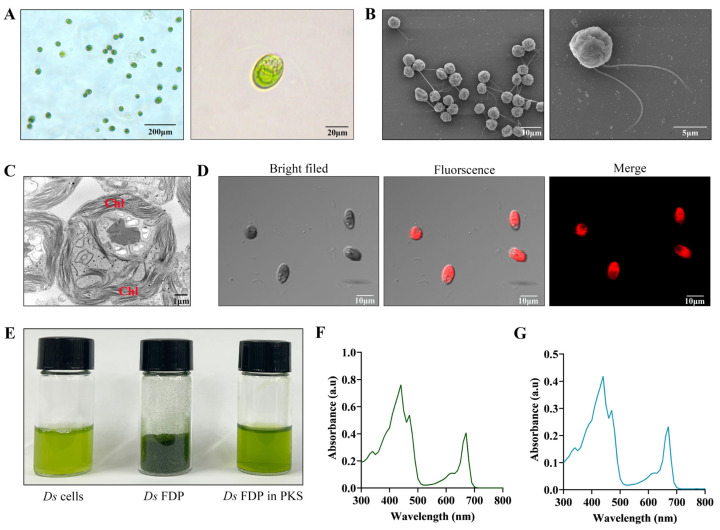
Characterization of *Ds* and *Ds* FDP. (**A**) The bright-field image of *Ds* cells. (**B**) High-resolution SEM image of *Ds* cells. (**C**) High-resolution TEM image of *Ds* cells (Chl: chloroplast). (**D**) Bright-field, fluorescence, and merge images of *Ds* cells. (**E**) *Ds* cells, *Ds* FDP, and appearance of *Ds* FDP suspension. (**F**,**G**) UV-vis spectra of *Ds* cells and *Ds* FDP.

Under a light microscope, *Ds* cells are green and spherical, and exhibit motility (Figure 1A). The SEM images revealed that *Ds* motility is due to the presence of a pair of flagella (Figure 1B), and TEM images showed that its green appearance results from abundant chloroplast (Figure 1C). Owing to the large chloroplast in *Ds*, it emits intense red fluorescent signals under appropriate excitation conditions without additional fluorescent markers, suggesting the autofluorescence characteristic of *Ds* (Figure 1D). To ensure long-term stability, *Ds* was lyophilized to form FDP. The resuspended *Ds* FDP in PKS exhibited a texture comparable to its pre-lyophilized state (Figure 1E). When compared the UV absorption spectra and ζ potential of the resuspended *Ds* FDP with those of the original live *Ds* cells, both samples exhibited two distinct absorption peaks, one at 430–450 nm and another at 650–670 nm (Figure 1F) [29]. Moreover, *Ds* FDP also exhibits the same dual UV absorption peaks (Figure 1G). Furthermore, ζ potential measurements showed that both *Ds* and *Ds* FDP particles possessed a similar surface charge (Figure S1A). The size stability of the *Ds*-FDP was assessed over 7 days. DLS analysis revealed that the Z-Average remained stable at 273.8 ± 2.04 nm. The corresponding polydispersity indices (PDIs) measured on days 1, 3, and 7 were 0.445, 0.401, and 0.452, respectively (Figure S4). Together, these data indicate that no significant particle aggregation or degradation occurred during the observation period. These results collectively demonstrate that the key characteristics of *Ds* were retained in the FDP.

### 3.2. Characterization of Dio

**Figure 2:**
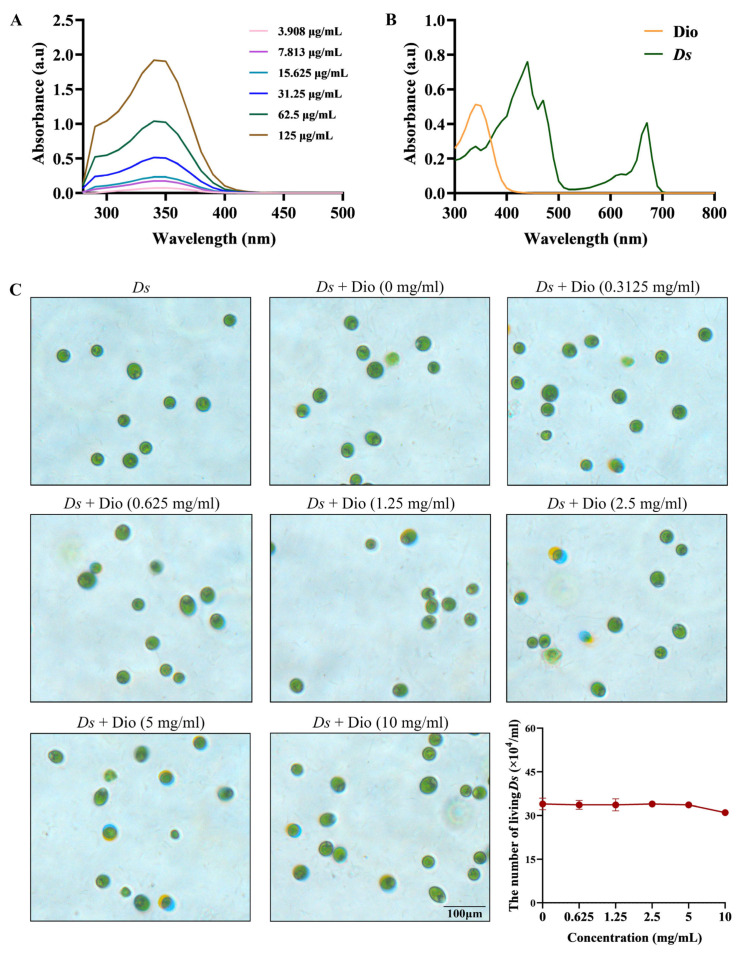
Characterization of Dio. (**A**) UV-vis spectra of different concentration Dio. (**B**) UV-vis spectra comparison of *Ds* cells and Dio. (**C**) Bright-field images of co-culture of *Ds* cells and series concentrations of Dio (Dio = 0, 0.3125, 0.625, 1.25, 2.5, 5, and 10 mg/mL) after 24 h, and the quantitative analysis of living *Ds* cells.

To establish a foundation for the drug delivery system, we first characterized the spectroscopic properties of the components. The optimal detection wavelength for Dio was determined to be 330 nm by full-wavelength scanning (Figure 2A). Critically, UV-Vis spectra confirmed distinct, non-overlapping absorption peaks between Dio and *Ds* carrier (Figure 2B), as well as between Dio and *Ds* FDP (Figure S1B). These distinct spectral profiles established the foundation for all subsequent analytical methods to quantify Dio concentration without interference. Subsequently, to determine a biocompatible concentration for co-culture, we evaluated the cytotoxicity of Dio on *Ds*. Results showed that *Ds* cells maintained good viability across all tested concentrations after 24 h (Figure 2C). However, a concentration of 5 mg/mL exhibited slight toxicity after 48 h (Figure S1C), thereby defining the upper safety threshold for subsequent experiments. In summary, the successful preparation and comprehensive characterization of both the *Ds* carrier and Dio drug have laid a solid foundation for the development of the subsequent drug delivery system.

### 3.3. Synthesis of Ds-Dio

**Figure 3:**
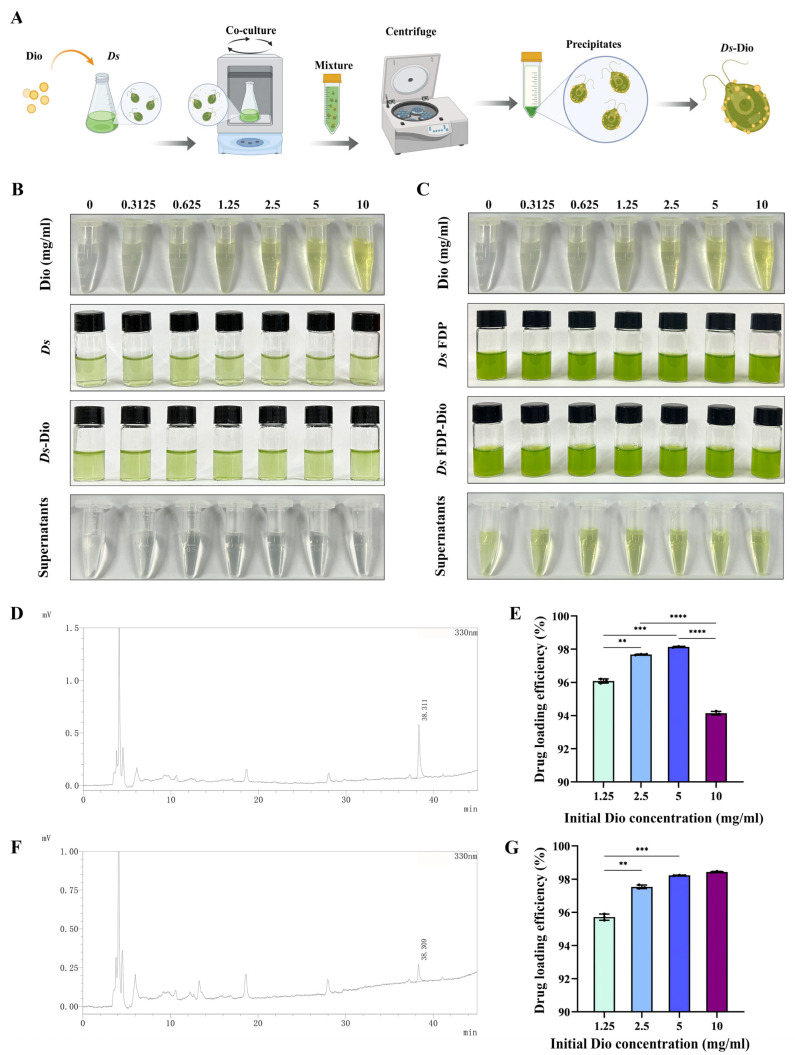
Synthesis and drug loading identification of *Ds*-Dio. (**A**) Schematic diagram of synthesis of *Ds*-Dio. (**B**,**C**) Photograph of the drug loading process of equivalent *Ds* cells and *Ds* FDP mixing with Dio at different concentrations. **(D**) The concentration of unloaded Dio in the supernatant after *Ds* loading Dio (Dio = 10 mg/mL) by HPLC. (**E**) Drug loading efficiency of *Ds* cells at initial Dio (Dio = 1.25, 2.5, 5, 10 mg/mL) concentrations. (**F**) The concentration of unloaded Dio in the supernatant after *Ds* FDP loading Dio (Dio = 10 mg/mL) by HPLC. (**G**) Drug loading efficiency of *Ds* FDP at initial Dio (Dio = 1.25, 2.5, 5, 10 mg/mL) concentrations (** *p* < 0.01 *** *p* < 0.001, **** *p* < 0.0001).

To develop a versatile and stable drug delivery platform, we utilized both live *Ds* cells and *Ds* FDP for loading Dio. This dual strategy allowed us to assess the fundamental drug-carrying efficiency of the algal biomass, while also evaluating the practicality of a storable, ready-to-use formulation. To synthesize the complexes, varying concentrations of Dio (0–10 mg/mL) were incubated with *Ds* or *Ds* FDP. After co-incubation, *Ds*-Dio complexes were collected by centrifugation (Figure 3A). The lack of a yellow color in supernatants indicated successful drug loading onto both carriers (Figure 3B,C). This was further corroborated by the ζ potential results, which showed that upon Dio binding, both *Ds* and *Ds* FDP shifted significantly toward more negative values (Figure S2A,B). Additionally, an increase in the particle size of the *Ds* FDP-Dio complex was observed (Figure S2C). To determine the drug loading efficiency, we first established an HPLC standard curve using a Dio reference substance (Figure S2D,E). The concentration of unbound Dio in the supernatant after co-incubation with *Ds* or *Ds* FDP was then quantified. For *Ds*, the residual Dio was measured after incubation with initial concentrations of 1.25, 2.5, 5, and 10 mg/mL (Figure S2F–H and Figure 3D), and it revealed that 5 mg/mL yielded the maximum loading efficiency (Figure 3E). Similarly, for *Ds* FDP, the residual Dio in the supernatant was measured (Figure 3F and Figure S2I–K), demonstrating high loading efficiency at both 5 and 10 mg/mL (Figure 3G). Consequently, 5 mg/mL was selected as the optimal concentration for preparing *Ds*-Dio in all subsequent experiments. The in vitro release study revealed that in simulated gastric fluid, free Dio exhibited a higher release rate compared to *Ds*-Dio and *Ds* FDP-Dio (Figure S5A). In contrast, within the intestinal fluid, *Ds*-Dio showed an increased release over both *Ds* FDP-Dio and free Dio (Figure S5B). These results indicate that the *Ds*-based carrier provides more effective control over the release of Dio under intestinal conditions. Therefore, based on the confirmed loading efficiency of both carriers, we selected the live *Ds*-Dio formulation for subsequent studies to harness the potential synergy between Dio and the biological activities of viable algal cells.

### 3.4. Ds-Dio Alleviates Cis-Induced AKI

**Figure 4:**
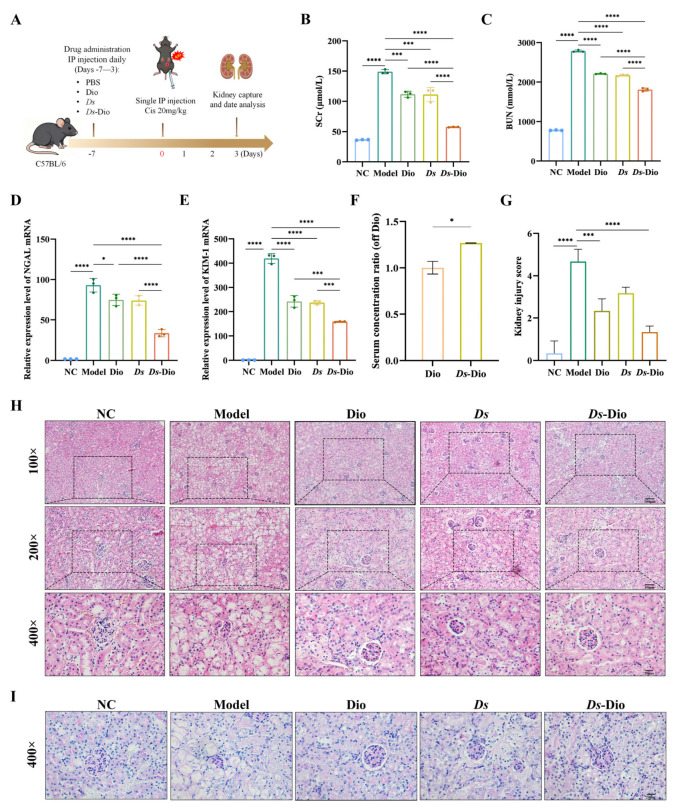
*Ds*-Dio alleviates the Cis-induced AKI. (**A**) Schematic illustration of a cisplatin-induced AKI mouse model. (**B**,**C**) Plasma Scr and BUN levels in mice. (**D**,**E**) Expression levels of acute kidney injury markers NGAL and KIM-1. (**F**) The plasma concentration of Dio in mice. (**G**,**H**) The corresponding glomerulus and tubular injury scores and representative HE-stained images of mouse renal tissue (100×, bar = 100 μm; 200×, bar = 50 μm; and 400×, bar = 20 μm). (**I**) Representative PAS-stained images of mouse renal tissue (400×, bar = 20 μm). The results are shown as the Mean ± SD (* *p* < 0.05, *** *p* < 0.001, **** *p* < 0.0001).

To evaluate the nephroprotective effect of *Ds*-Dio, we established a cisplatin-induced mouse model of AKI (Figure 4A). Successful model induction was confirmed by a significant increase in Scr and BUN levels in the model group compared to the normal group. Treatment with Dio or *Ds* alone significantly reduced these levels, while the *Ds*-Dio combination group exhibited a more pronounced therapeutic effect (Figure 4B,C). Consistent with this, the expressions of kidney injury markers KIM-1 and NGAL were attenuated by Dio and *Ds*, with the greatest reduction observed in the *Ds*-Dio group (Figure 4D,E). To investigate the mechanism underlying the enhanced efficacy of *Ds*-Dio, we measured the plasma concentration of Dio. The results showed a significantly higher Dio level in the *Ds*-Dio group than in the Dio-alone group, indicating improved bioavailability of Dio when delivered via the *Ds* carrier (Figure 4F). Furthermore, the quantitative injury score further confirmed the therapeutic effects of Dio and *Ds*, as well as the superior combined effect of *Ds*-Dio (Figure 4G). Histological examination by HE staining also revealed severe renal pathology in the model group, including edema, inflammatory cell infiltration, and glomerular and tubular damage. These pathological changes were markedly ameliorated in the Dio, *Ds*, and *Ds*-Dio treatment groups (Figure 4H). A similar trend was observed in PAS staining (Figure 4I). In conclusion, these results demonstrated that Dio alleviates cisplatin-induced AKI, and this protective effect can be enhanced by the *Ds*-based delivery system through improved bioavailability of Dio.

The biosafety of *Ds*-Dio drug delivery system was systematically evaluated in the AKI mouse model. During the pretreatment period before modeling, body weight of mice in the Dio, *Ds*, and *Ds*-Dio groups maintained stable growth (Figure S3A). Following cisplatin administration to induce AKI, a characteristic decrease in body weight was observed in the model group, as expected (Figure S3A). This weight loss was also evident, to a comparable extent, in the Dio, *Ds*, and Ds-Dio treatment groups, suggesting it was a consequence of the disease model itself rather than a toxic effect of the treatments. Assessment of organ index revealed a significant increase in the spleen index in the Dio, *Ds*, and *Ds*-Dio groups compared to the control and model groups (Figure S3B,C). This suggests a systemic immunomodulatory effect induced by the *Ds*-based delivery system. HE staining demonstrated no obvious morphological alterations in vital organs such as the liver across all five groups, including those receiving the combined *Ds*-Dio formulation (Figure S3E). Collectively, these findings demonstrate that *Ds*-Dio exhibits a favorable safety profile in vivo, with no evidence of overt toxicity to major organs such as the liver (Figure S3D).

### 3.5. Effects of Dio on AKI Based on Network Pharmacology

**Figure 5:**
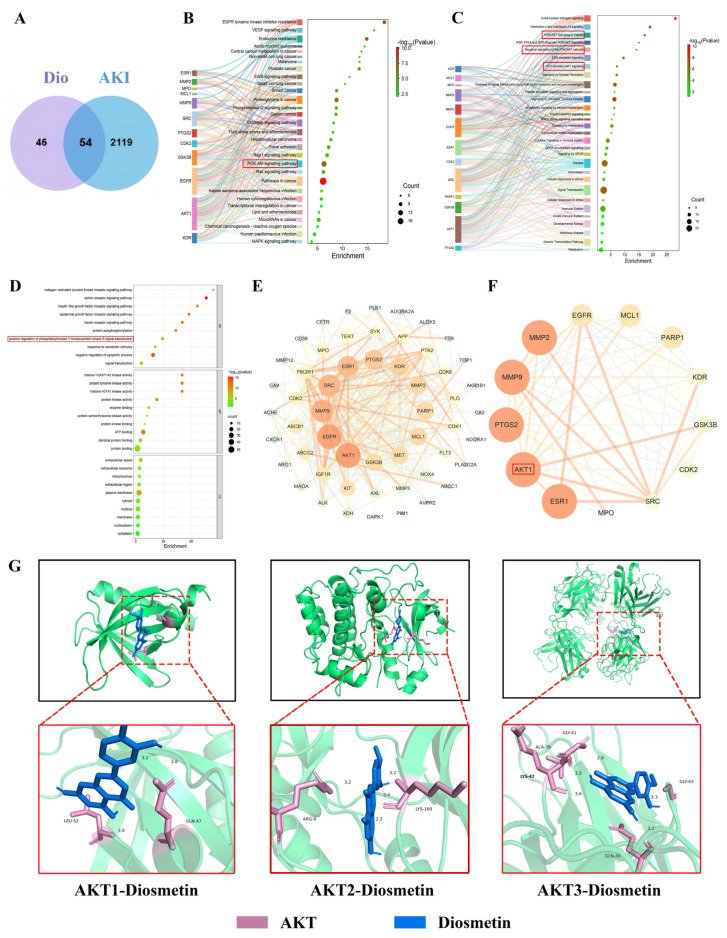
Effects of Dio on AKI based on network pharmacology. (**A**) Venn diagram of potential therapeutic targets of Dio for AKI. (**B**) KEGG signaling pathway analysis. (**C**) Reactome enrichment analysis. (**D**) GO enrichment analysis. (**E**) PPI network analysis. (**F**) The core genes of PPI network. (**G**) Molecular docking between Dio and AKT.

To systematically investigate the therapeutic mechanism of Dio against AKI, we performed an integrated network pharmacology analysis. Potential targets of Dio were identified from the PubChem and Swiss Target Prediction databases, yielding 100 candidates. Meanwhile, 2173 AKI-related targets were obtained from the Gene Cards and OMIM databases. A Venn diagram identified 54 overlapping genes as potential therapeutic targets of Dio for AKI (Figure 5A). Subsequent KEGG pathway enrichment analysis of these targets highlighted the significant involvement of the PI3K/AKT signaling pathway (Figure 5B); this finding was further supported by both Reactome and GO biological process enrichment analyses (Figure 5C,D). To further refine these insights, a protein–protein interaction (PPI) network was constructed, from which 13 core targets were distilled, with AKT1 emerging as a key nodal protein within the PI3K/AKT cascade (Figure 5E,F). Importantly, molecular docking simulations confirmed that Dio forms stable binding interactions with key residues on AKT isoforms: AKT1 (LEU-52, GLN-47), AKT2 (ARG-6, LYS-160), and AKT3 (LYS-42, ALA-39, GLY-41, GLY-43, GLN-40) (Figure 5G). Collectively, this multi-layered evidence indicates that Dio alleviates AKI primarily by modulating the PI3K/AKT signaling pathway, with AKT serving as its direct molecular target.

### 3.6. Ds-Dio Reduces the Oxidative Stress and Inflammation Regulated via PI3K/AKT in AKI

**Figure 6:**
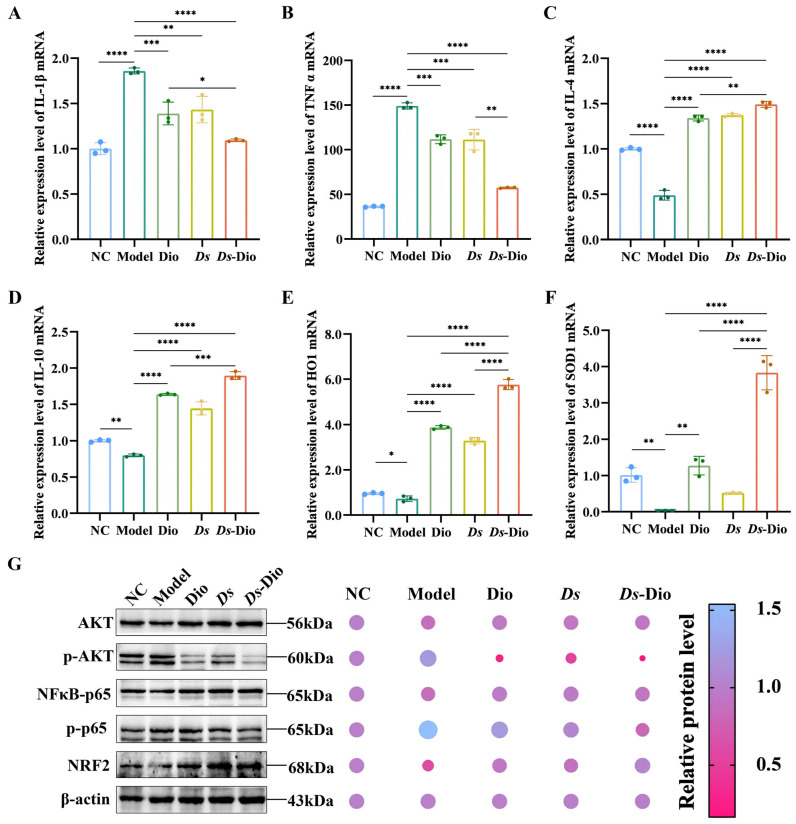
*Ds*-Dio reduces the oxidative stress and inflammation regulated via PI3K/AKT in AKI. (**A**–**F**) The relative expression levels of TNF-α, IL-1β, IL-4, IL-10, SOD1, and HO1 mRNA in renal tissues. (**G**) Western blotting images of indicated protein expression in renal tissues. The results are shown as the Mean ± SD (* *p* < 0.05; ** *p* < 0.01, *** *p* < 0.001, **** *p* < 0.0001).

Previous studies have established the critical role of inflammation and oxidative stress in cisplatin-induced nephrotoxicity [30]. To evaluate the anti-inflammatory effects of our interventions, we measured key pro- and anti-inflammatory cytokines in renal tissues. The model group showed significantly elevated levels of TNF-α and IL-1β, which were markedly suppressed by Dio, *Ds*, and most effectively by the *Ds*-Dio combination (Figure 6A,B). Concurrently, *Ds*-Dio significantly upregulated the anti-inflammatory cytokines IL-4 and IL-10 (Figure 6C,D). In parallel, oxidative stress markers were assessed. The model group exhibited downregulated expression of the antioxidant enzymes SOD1 and HO1, which was partially reversed by Dio or *Ds* alone, and more robustly restored by the *Ds*-Dio combination (Figure 6E,F). Given the recognized role of the PI3K/AKT pathway in regulating both NF-κB-driven inflammation and NRF2-mediated antioxidant responses, we investigated its involvement. Western blot analysis revealed that cisplatin-induced AKI enhanced phosphorylation of AKT and p65, indicating PI3K/AKT/NF-κB pathway activation. Both Dio and *Ds*-Dio treatments reduced this phosphorylation, with the combination showing superior inhibition (Figure 6G). Conversely, NRF2 expression was suppressed in the model group but was effectively restored by Dio and, to a greater extent, by *Ds*-Dio (Figure 6G). In summary, the *Ds*-Dio combination exerts a synergistic nephroprotective effect by co-modulating the PI3K/AKT pathway, resulting in the dual inhibition of NF-κB-mediated inflammation and activation of NRF2-dependent antioxidant response.

## 4. Discussion

Regarding the treatment of AKI, the integrated approaches incorporating TCM have currently gained increasing attention and shown promising potential in mitigating renal injury, highlighting a viable pathway for novel drug development [31]. Among the various experimental models of AKI, including ischemia–reperfusion, ureteral obstruction, and drug-induced injury, the cisplatin-induced model is particularly clinically relevant [32]. Cisplatin, a first line chemotherapeutic agent for solid tumors, is dose-limited by its severe nephrotoxicity, which frequently leads to treatment interruption or discontinuation, thereby compromising oncological outcomes [1]. Given this clinical dilemma, we selected the cisplatin-induced AKI model to evaluate potential nephroprotective agents that could be co-administered during cisplatin-based chemotherapy. This approach not only allows for the exploration of TCM-derived compounds in AKI intervention, but also aligns with the urgent need to mitigate chemotherapy-induced organ damage without compromising antitumor efficacy. Dio possesses diverse pharmacological properties, including anti-inflammatory, antibacterial, antioxidant, and antitumor activities [33,34]. While previous work suggested that Dio could mitigate cisplatin-induced renal injury, these studies used a cisplatin dosage insufficient to induce significant AKI, leaving its nephroprotective efficacy unvalidated. Here, using a well-established cisplatin-induced AKI mouse model, we definitively demonstrated that Dio treatment ameliorated renal dysfunction, as indicated by reduced Scr and BUN levels. It also suppressed the expression of tubular injury biomarkers (KIM-1 and NGAL) and attenuated histopathological damage as shown in HE- and PAS-stained tissues [35,36]. Our findings provide direct and compelling evidence for the therapeutic potential of Dio against cisplatin-AKI. Notably, Dio is not an isolated case among TCM-derived compounds in exerting nephroprotective effects. For instance, baicalein, a major bioactive flavonoid from *Scutellaria baicalensis Georgi*, was recently shown to alleviate cisplatin-induced AKI by inhibiting ALOX12 dependent ferroptosis [37]. Similarly, paeoniflorin, the principal active component of *Paeonia lactiflora Pall*, protects against cisplatin-induced AKI by targeting the Hsp90AA1-AKT protein–protein interaction [5]. Together with our findings on Dio, these examples underscore the substantial clinical potential of TCM in prevention and treatment of AKI.

As a flavonoid, Dio suffers from poor aqueous solubility and suboptimal chemical stability, which directly impairs its oral absorption and therapeutic potential, thus limiting its long-term clinical application. To overcome these challenges, strategies such as structural optimization and advanced drug delivery systems have been proposed to enhance its bioavailability and efficacy [33]. And others reported that encapsulating Dio in zein complexes modified with galactose and rhamnogalacturonan-I rich pectin significantly enhances its stability and targeting capacity [38]. Therefore, the development of a novel delivery platform represents a viable and necessary strategy to improve the pharmacokinetic profile of Dio and accelerate its clinical translation. In this study, we have developed a novel Dio delivery system based on microalgae *Ds*. *Ds* presents several inherent advantages as a drug carrier, primarily due to its unique cellular structure. Unlike most plant cells, *Ds* lacks a rigid cell wall and is enclosed only by a flexible plasma membrane rich in various membrane proteins, such as ion channels and proton pumps [39]. This structural feature confers remarkable membrane plasticity, allowing the cell to dynamically adjust its surface area in response to osmotic changes without losing integrity [40]. Observations by SEM confirmed that although the cell membrane appears wrinkled, it remains structurally continuous. TEM further revealed an abundance of chloroplasts, which confer natural red autofluorescence, which is a valuable property for tracking the carrier without external labeling. In addition, the large surface area, rich membrane protein content, and negatively charged zeta potential of *Ds* collectively facilitate efficient substance adsorption. Beyond its physicochemical advantages, *Ds* has been widely recognized for its biosafety, non-toxicity, and edible properties, and has demonstrated intrinsic biological activities including anti-inflammatory, antioxidant and antitumor effects [21,22,41,42,43]. These multifunctional attributes are seldom available in conventional synthetic nanocarriers. Importantly, the viability of *Ds* cells remained unchanged after 24 h of co-culture with Dio at working concentrations, indicating no significant cytotoxicity from the drug. Based on these properties, we employed *Ds* as a natural delivery vehicle to construct the *Ds*-Dio system. To further assess the versatility of this platform, we evaluated the characteristics both live *Ds* cells and their freeze-dried powder. After being loaded by *Ds* or *Ds* FDP carriers, the release behavior of Dio was assessed. Both carriers protected Dio from acidic degradation in the stomach and effectively controlled as well as enhanced its release in the intestine. Notably, the *Ds* carrier provided superior control over the release kinetics compared to the *Ds* FDP. In addition, the *Ds* FDP formulation demonstrated retained drug loading efficiency and size stability, highlighting the robustness of the system and its potential for storage and distribution.

Following the successful establishment of the *Ds*-Dio drug delivery system, we evaluated its therapeutic efficacy in a mouse model of AKI. In addition, to evaluate whether *Ds* or *Ds*-Dio affects the antitumor efficacy of Cis, we conducted cell proliferation assays across various cancer cell lines. Notably, the results demonstrated that *Ds* or *Ds*-Dio did not compromise cisplatin’s efficacy and can enhance its anti-proliferative activity in a cell-context-dependent manner. However, the enhanced anti-proliferative effect of *Ds*-Dio combined with cisplatin was significant in the human cell lines HCT116 and DLD-1, but markedly less so in the mouse-derived CT26 line (Figure S6A–C). Differences in the interaction between the micrometer-sized *Ds* carrier and the surface properties of various cell lines could affect the cellular uptake of Dio. Furthermore, the intrinsic molecular context-such as divergent signaling pathways, mutation profiles, proliferation rates, and baseline sensitivity to flavonoids may contribute to the cellular response to the combination therapy. Animal experiment results clearly showed that *Ds*-Dio drug delivery system was more effective than Dio alone in treating kidney injury. This enhanced efficacy can be attributed to improved drug delivery. Moreover, mice treated with *Ds*-Dio showed a significantly higher serum concentration of Dio than those receiving Dio alone, confirming that *Ds* carrier markedly enhances the bioavailability of Dio. An interesting observation was that the strong anti-AKI effect of *Ds*-Dio was accompanied by a noticeable increase in spleen size. We interpret this splenomegaly not as an adverse effect, but rather as a sign of a beneficial, systemically enhanced immune response. As a natural biological entity, the *Ds* carrier likely acts as an immune adjuvant, boosting the body’s own defense mechanisms. The spleen, a key organ for immune cell activity, may expand temporarily due to increased efforts to resolve inflammation and repair tissue damage in AKI. *Ds* offers unique biological properties and the large size of *Ds* promotes phagocytic clearance, which is consistent with our observation of splenomegaly in *Ds*-treated mice. This primarily reflects the expected clearance pathway via the mononuclear phagocyte system (e.g., in the spleen), which can prevent long-term circulation and offer a natural degradation route post-delivery. Beyond size, its flagella confer natural motility, potentially enhancing distribution, and as a living cell, *Ds* provides intrinsic antioxidant and anti-inflammatory bioactivity that may synergize with Dio. Therefore, the spleen enlargement further supports the enhanced immunomodulatory capacity of *Ds*-Dio formulation, which contributes to its superior therapeutic outcome. We will further investigate the specific mechanisms underlying this phenomenon in subsequent studies.

Mechanistically, the network pharmacology analysis identified the PI3K/AKT signaling pathway as a central mechanism through which Dio may exert its effects in AKI. This computational finding provided a strong mechanistic hypothesis, which we subsequently sought to validate experimentally. Given the well-established roles of inflammation and oxidative stress in the pathogenesis of AKI [44,45], and considering that PI3K/AKT pathway has been widely reported to regulate both NF-κB-mediated inflammatory responses and NRF2-driven antioxidant defense [38,46], we first investigated whether the protective effects of Dio and *Ds*-Dio were associated with the amelioration of these two pathological processes. Our results demonstrated that *Ds*-Dio treatment significantly modulated key biomarkers of inflammation and oxidative stress. As expected, the formulation effectively downregulated pro-inflammatory cytokines (IL-1β and TNF-α) and upregulated anti-inflammatory cytokines (IL-4 and IL-10). Simultaneously, it restored the expression of the antioxidant enzymes SOD1 and HO1. These findings confirmed that protective effects of *Ds*-Dio are indeed mediated through concurrent anti-inflammatory and antioxidant actions. To further dissect the upstream regulatory mechanism and directly test the prediction from network pharmacology, we examined the activity of PI3K/AKT pathway and its downstream effectors NF-κB and NRF2. The Western blot analysis revealed that *Ds*-Dio treatment significantly suppressed the phosphorylation of AKT and NF-κB p65, while markedly enhancing the protein expression of NRF2. This result provides direct experimental evidence that Dio, particularly when delivered via the *Ds* carrier, alleviates AKI by modulating the PI3K/AKT pathway, which in turn coordinately inhibits the NF-κB mediated inflammatory cascade and activates the NRF2-dependent antioxidant response. In summary, our study establishes a coherent mechanistic pathway from computational prediction to functional validation. We demonstrate that the *Ds*-Dio combination synergistically protects against cisplatin-induced nephrotoxicity by targeting the PI3K/AKT axis to concomitantly mitigate inflammation and oxidative stress, thereby offering a promising multi-target therapeutic strategy for AKI.

## 5. Conclusions

In conclusion, this study successfully developed Ds-Dio, a microalgae-based delivery system that enhances the nephroprotective efficacy of Dio against cisplatin-induced AKI. More importantly, this work elucidates that this protection is associated with modulation of the PI3K/AKT pathway, which concurrently suppresses NF-κB-mediated inflammatory responses and NRF2-regulated oxidative stress. Beyond improving the bioavailability of Dio, our findings highlight the dual role of Ds as both a natural therapeutic agent and an efficient drug carrier. This work not only provides a promising strategy for mitigating cisplatin-induced nephrotoxicity, but also supports the broader application of microalgae-based systems in drug delivery for enhanced treatment of AKI and other inflammatory-oxidative stress-related diseases.

## Data Availability

The data that support the findings of this study can be obtained from the first author and corresponding author.

## Supplementary Materials

The following are available online at https://www.mdpi.com/article/10.3390/pharmaceutics18010102/s1, Table S1: The primer sequences of qRT-PCR, Figure S1: Characteristics of Dio, Figure S2: Synthesis and characterization analysis of Ds-Dio, Figure S3: Safety index detection in vivo. Figure S4: Size stability assessment of Ds-FDP, Figure S5: In vitro the release percentage variation of Dio, Figure S6: Cell proliferation changes in various cancer cell lines over time.
