# Supplementary material for: Dunaliella salina-Loaded Diosmetin Carriers Alleviate Oxidative Stress and Inflammation in Cisplatin-Induced Acute Kidney Injury via PI3K/AKT Pathway
Source: Pharmaceutics. 2026 Jan 12;18(1):102. doi: 10.3390/pharmaceutics18010102 (PMC12844676; doi:10.3390/pharmaceutics18010102)
Supplement: Supplementary file 1 [file pharmaceutics-18-00102-s001.zip › pharmaceutics-4037684-supplementary.pdf]

Supplementary Materials and methods

Cell lines

The human colon adenocarcinoma cells DLD1 and HCT116, the mouse colon adenocarcinoma cells CT26 were originally obtained from the Cell Bank of the Chinese Academy of Sciences (Shanghai, China). CT26 and DLD1 cells were cultured in RPMI-1640 medium (Biological Industries, Israel). HCT116 cells were cultured in McCoy's 5A medium (Biological Industries, Israel). Both culture medium was supplemented with containing10% (v/v) fetal bovine serum and 1% (v/v) penicillin/streptomycin. All cells were maintained in an incubator containing 5% CO_2 and 95% relative humidity at 37 °C.

Size stability assessment of *Ds*-FDP

The prepared *Ds*-FDP was re-suspended in fresh PKS medium and subsequently diluted with PBS. This suspension was stored at 4 °C to assess its size stability. Measurements were performed directly on the stored suspension on days 1, 3, and 7 without further processing. The mean particle size (Z-average) and the polydispersity index (PDI) of the *Ds*-FDP stored suspension were measured via DLS for each time point.

In vitro the release percentage variation of Dio

Simulated gastric fluid (pH = 1.2) and simulated intestinal fluid (pH = 6.8) were purchased from Shanghai Macklin Biochemical Technology Company (Shanghai, China). The prepared Dio, *Ds*-Dio, and *Ds* FDP-Dio were resuspended in 5 mL of simulated gastric fluid (pH = 1.2) or simulated intestinal fluid (pH = 6.8), respectively, and stirred at 37°C. At each the scheduled time point (0.5, 1, 1.5, 2, 4, 6, 8, 10, 12, 24h), a 2 mL sample of the suspension was centrifuged at 3000g for 30 min to obtain the supernatant. And the supernatant was tested at 330 nm by HPLC, in combination with a standard curve of Dio. Meanwhile, the suspension was replaced with an equal volume of fresh simulated fluid.

Cell proliferation assay

Cells were seeded in 96-well plates and incubated at 37 °C overnight. The numbers of cells in 96-well plate were 6000/well. Different concentrations of drugs (Cis: 10 μM, Cis+*Ds,* and Cis+*Ds*-Dio: loaded with 40 μM Dio) were added to the culture medium. Cells were cultured for 24 h and 48 h respectively for proliferation assay. After removing the 96-well plates from the incubator, the cells were fixed with 4% paraformaldehyde solution at room temperature for 5 min. Then, stained with 0.1% crystal violet at 37°C for 5 min. Following staining, cells were washed, air-dried, and then dissolve in 1% SDS. Absorbance was then measured at 595 nm using a microplate reader.

Table S1. The primer sequences of qRT-PCR

GAPDH	Forward	5'-AGGTCGGTGTGAACGGATTTG-3'
	Reverse	5'-TGTAGACCATGTAGTTGAGGTCA-3'
NGAL	Forward	5'-TGGCCCTGAGTGTCATGTG-3'
	Reverse	5'-CTCTTGTAGCTCATAGATGGTGC-3'
KIM-1	Forward	5'-GTTAAACCAGAGATTCCCACACG-3'
	Reverse	5'-TCTCATGGGGACAAAATGTAGTG-3'
IL-1β	Forward	5'-GCAACTGTTCCTGAACTCAACT-3'
	Reverse	5'-ATCTTTTGGGGTCCGTCAACT-3'
TNF-α	Forward	5'-CCCTCACACTCAGATCATCTTCT-3'
	Reverse	5'-GCTACGACGTGGGCTACAG-3'
IL-4	Forward	5'-GGTCTCAACCCCCAGCTAGT-3'
	Reverse	5'-GCCGATGATCTCTCTCAAGTGAT-3'
IL-10	Forward	5'-GCTCTTACTGACTGGCATGAG-3'
	Reverse	5'-CGCAGCTCTAGGAGCATGTG-3'
SOD1	Forward	5'-AACCAGTTGTGTTGTCAGGAC-3'
	Reverse	5'-CCACCATGTTTCTTAGAGTGAGG-3'
HO1	Forward	5'-TGCAGGTGATGCTGACAGAGG-3'
	Reverse	5'-GGGATGAGCTAGTGCTGATCTGG-3'

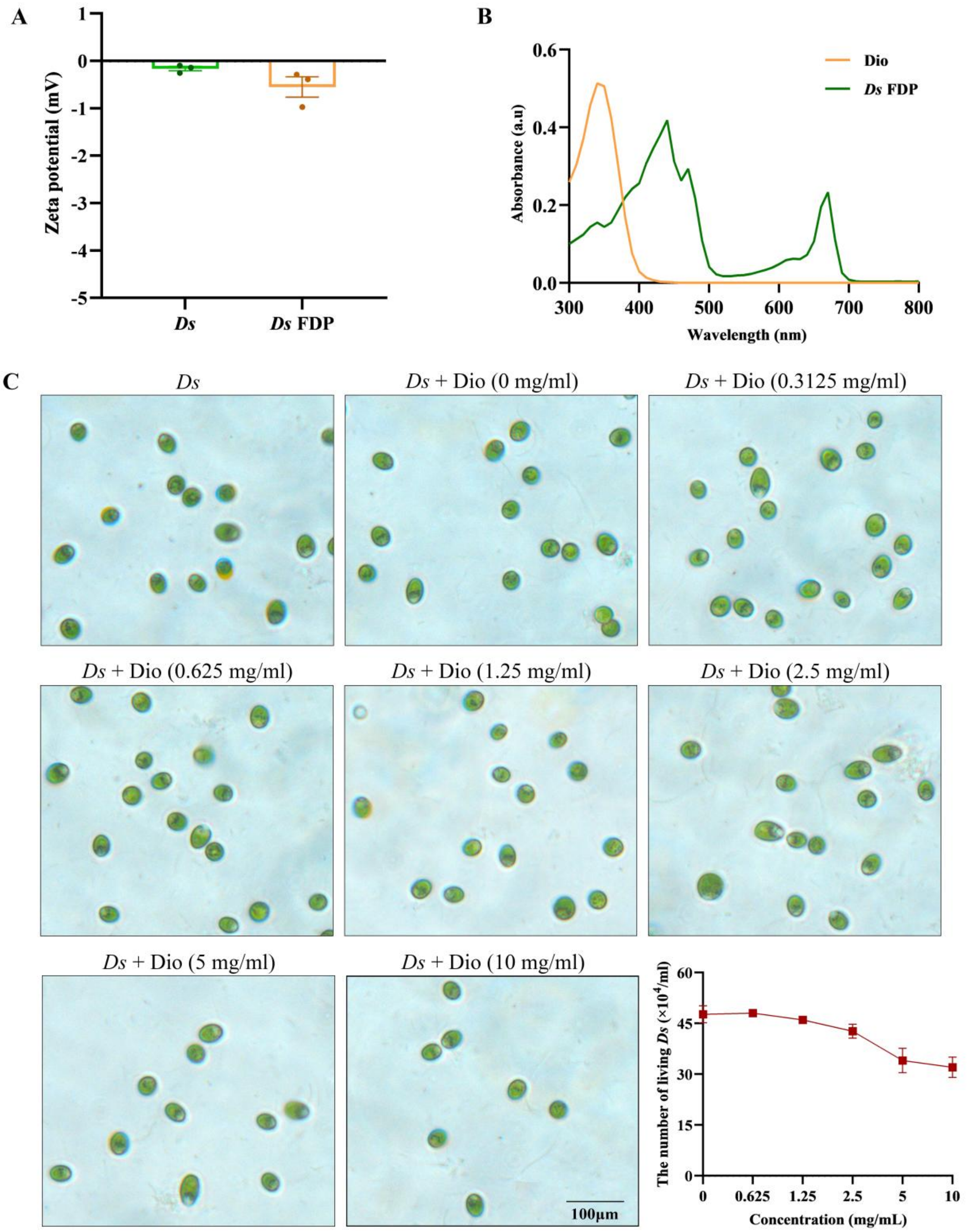

Fig. S1 Characteristics of Dio.

(A) The ζ potential of *Ds* and *Ds* FDP. (B) UV-vis spectra comparison of *Ds* FDP and Dio. (C) Bright-field images of co-culture of *Ds* cells and series concentrations of Dio (Dio = 0, 0.3125, 0.625, 1.25, 2.5, 5, and 10 mg/mL) after 48 h, and the quantitative analysis of living *Ds* cells.

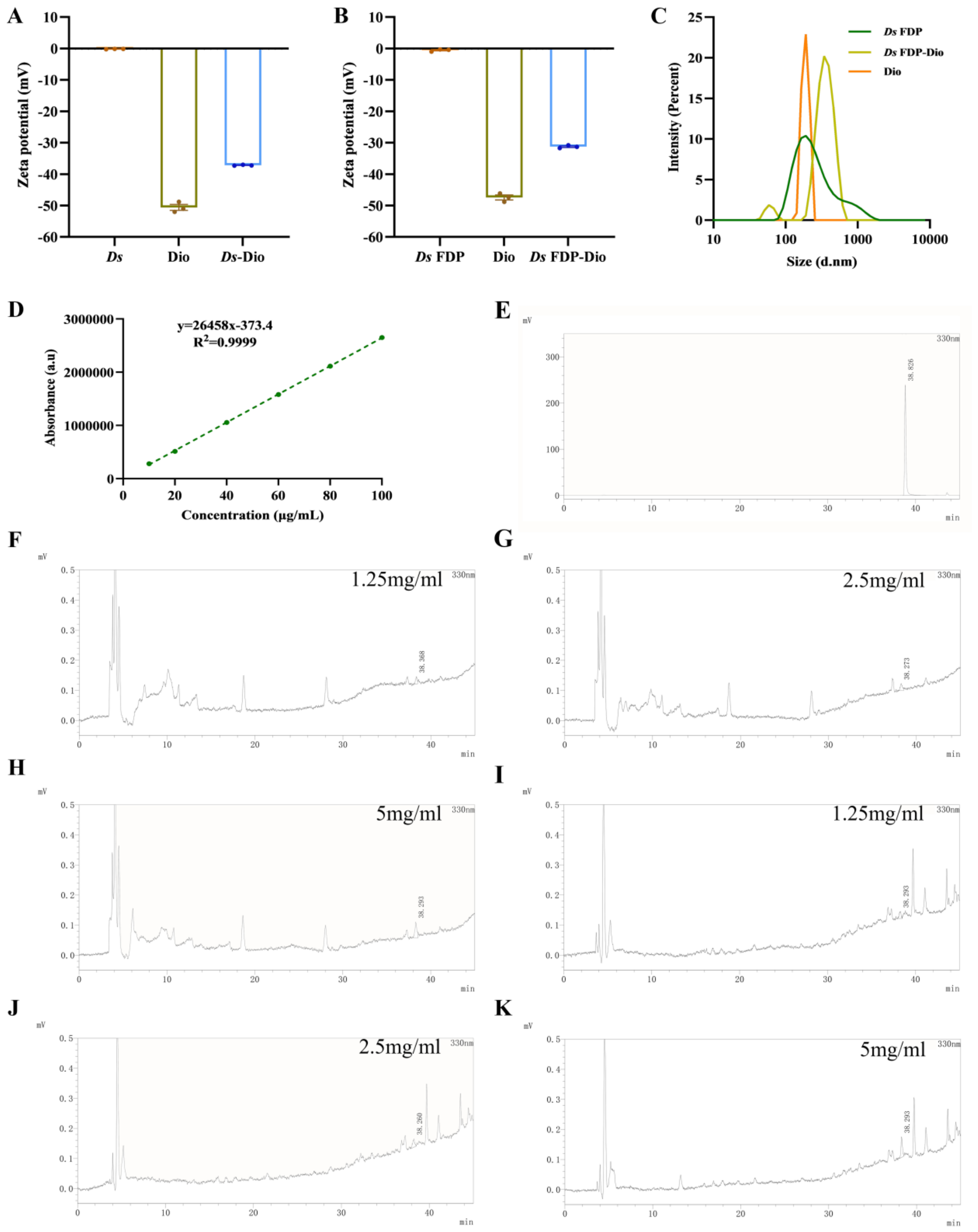

Fig. S2 Synthesis and characteriaztion analysis of *Ds*-Dio.

(A-B) Changes in ζ potential of *Ds* and *Ds* FDP before and after loading Dio. (C) The particle size of *Ds* FDP, Dio and *Ds* FDP-Dio. (D) HPLC standard curve of Dio reference substance. (E) HPLC chromatogram of Dio reference substance. (F-H) The concentration of unloaded Dio in supernatant after *Ds* loading Dio (Dio = 1.25, 2.5, 5 mg/mL) by HPLC. (I-K) The concentration of unloaded Dio in supernatant after *Ds* FDP loading Dio (Dio = 1.25, 2.5, 5 mg/mL) by HPLC.

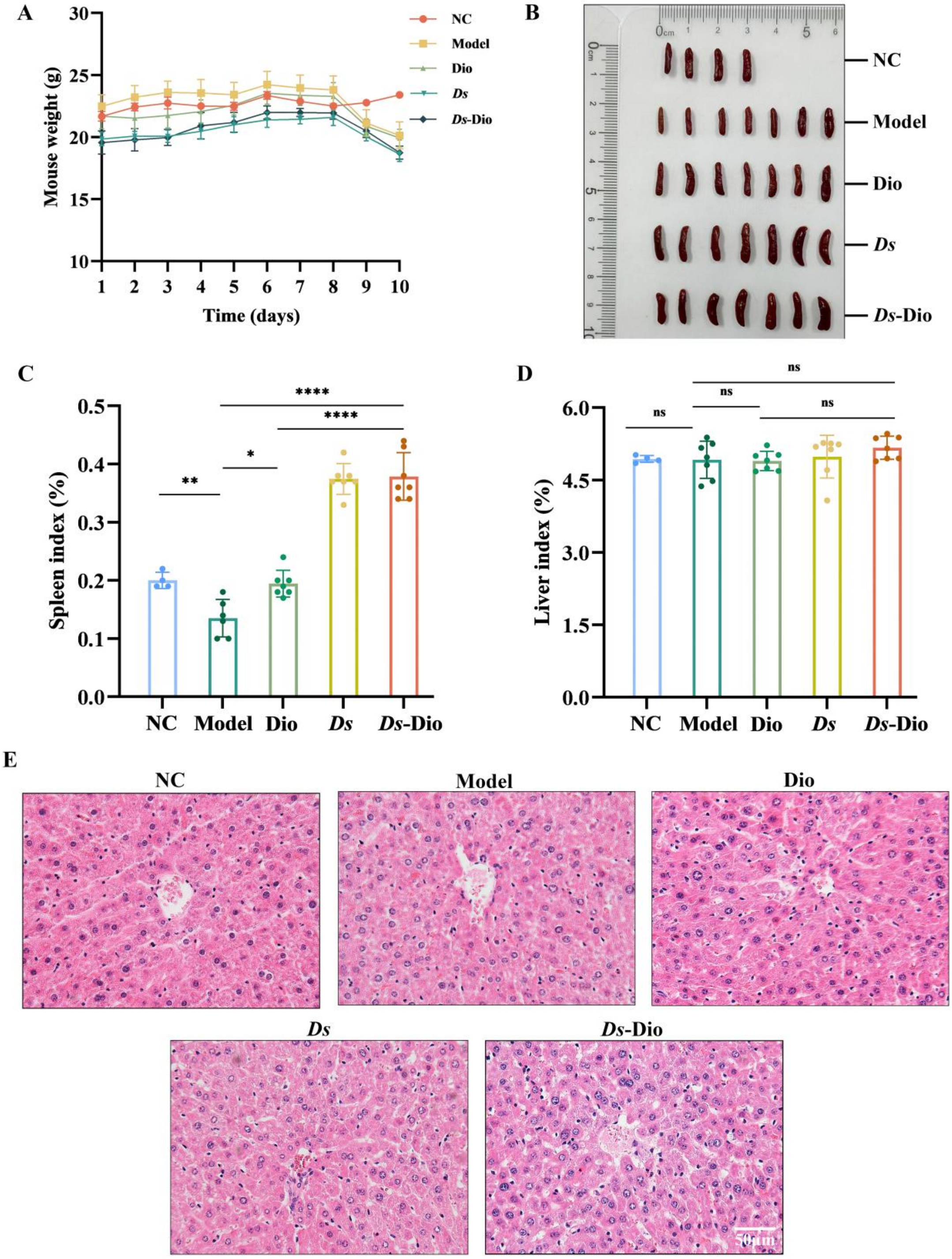

Fig. S3 Safety index detection in vivo.

(A) Body weight change of mice in different groups. (B-C) Pictures of mouse spleens and spleen index in different groups. (D) Liver index in different groups. (E) Representative HE-stained images of mouse vital organs in different groups (scale bar = 50 μm). The results are shown as the Mean ± SD ($^{*}P$<0.05; $^{**}P$<0.01; $^{****}P$<0.0001).

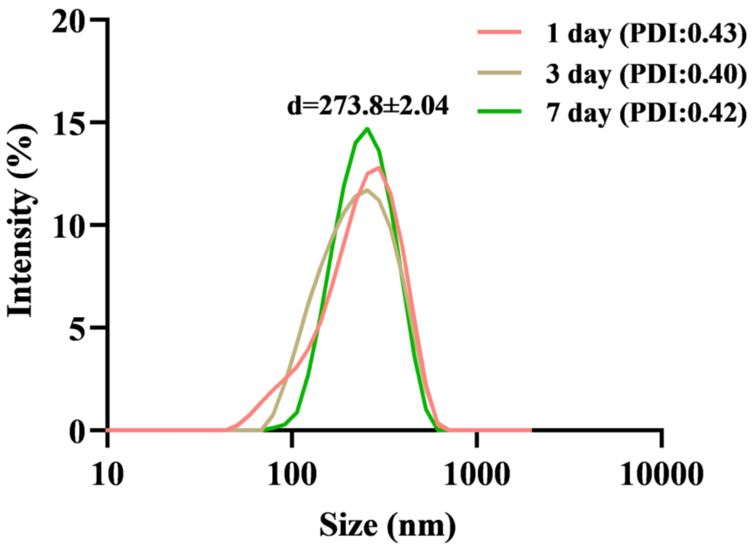

Fig. S4 Size stability assessment of *Ds*-FDP.

Dynamic light scattering (DLS) intensity distributions measured on days 1, 3, and 7, with corresponding polydispersity indices (PDI) of 0.43, 0.40, and 0.42.

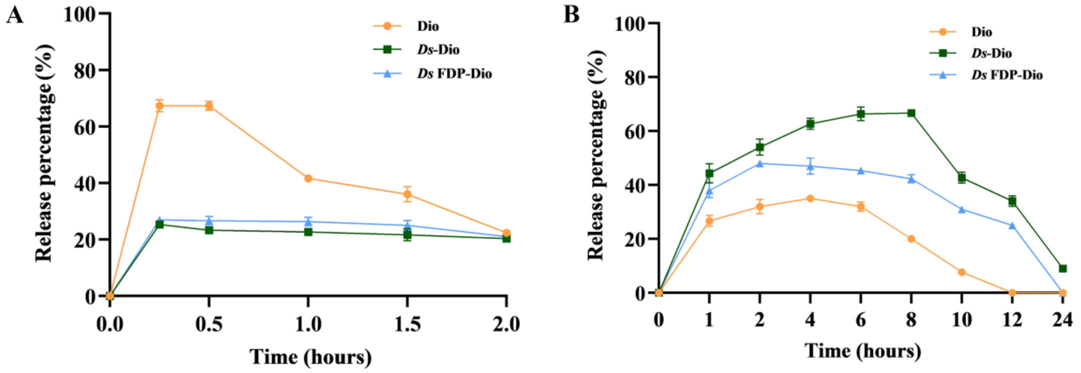

Fig. S5 In vitro the release percentage variation of Dio.

(A) Release percentage of different forms of Dio at simulated gastric fluid (pH = 1.2) over various time points. (B) Release percentage of different forms of Dio at simulated intestinal fluid (pH = 6.8) over various time points.

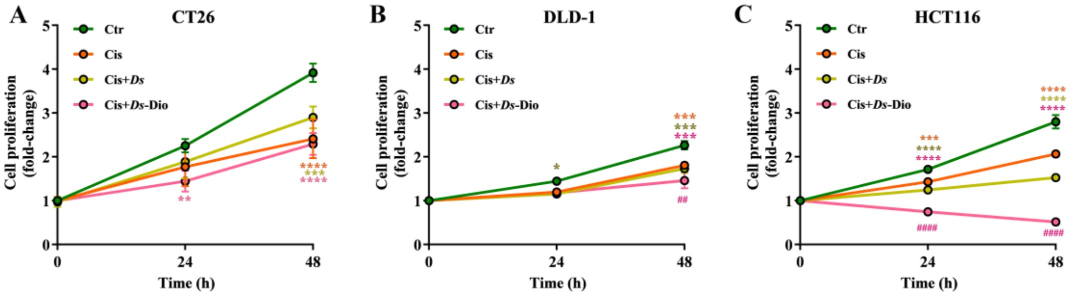

Fig. S6 Cell proliferation changes in various cancer cell lines over time.

(A) Proliferation changes of CT26 cells treated with Cis, Cis+*Ds*, or Cis+*Ds*-Dio for 24 and 48 hours. (B) Proliferation changes of DLD1 cells treated with Cis, Cis+*Ds*, or Cis+*Ds*-Dio for 24 and 48 hours. (C) Proliferation changes of HCT116 cells treated with Cis, Cis+*Ds*, or Cis+*Ds*-Dio for 24 and 48 hours. * represents different groups vs. Ctr group, # represents Cis+*Ds*-Dio group vs. Cis group. The results are shown as the Mean±SD ($^{*}P$<0.05; $^{**}P$<0.01; $^{***}P$<0.001; $^{****}P$<0.0001; $^{\#\#}P$<0.01; $^{\#\#\#\#}P$<0.0001).
